# Biostimulators: A New Trend towards Solving an Old Problem

**DOI:** 10.3389/fpls.2016.00748

**Published:** 2016-05-31

**Authors:** Małgorzata M. Posmyk, Katarzyna Szafrańska

**Affiliations:** Department of Ecophysiology and Plant Development, Faculty of Biology and Environmental Protection, University of LodzLodz, Poland

**Keywords:** bioregulators, biostimulants, environmental stresses, organic farming, plant stress physiology

## Abstract

Stresses provoked by adverse living conditions are inherent to a changing environment (climate change and anthropogenic influence) and they are basic factors that limit plant development and yields. Agriculture always struggled with this problem. The survey of non-toxic, natural, active substances useful in protection, and stimulation of plants growing under suboptimal and even harmful conditions, as well as searching for the most effective methods for their application, will direct our activities toward sustainable development and harmony with nature. It seems highly probable that boosting natural plant defense strategies by applying biostimulators will help to solve an old problem of poor yield in plant cultivation, by provoking their better growth and development even under suboptimal environmental conditions. This work is a concise review of such substances and methods of their application to plants.

## Introduction

All living organisms including plants may be frequently exposed to adverse environmental conditions. Different intensity and duration of a negative stimulus generate stress, which triggers specific or non-specific reaction. The former usually appears when a stressor focuses on a particular target, the latter when reactions to different stressors are similar. Since plants being rooted cannot escape from a harmful environment, they evolved other defense strategies including (i) ability to avoid stress (*i.a.*, morphological and biochemical barriers preventing or delaying stressor activity inside a cell, adaptation of a life cycle to seasons) and/or (ii) stress tolerance (*i.a*. alternative pathways allowing a cell to function under stressful conditions, prevention of stress-generated changes, tolerance of changes or mechanisms of fast damage repair).

It is clear that crop plant production methods based only on improving agricultural technology (e.g., tillage, re-cultivation, fertilization, irrigation, etc.) are limited due to the inability to effectively use the biological potential of the cultivated variety. In the face of a difficult task of preventing damage caused by harmful organisms or abiotic stresses in field crops, the plant production and protection should be based on stimulation of their growth and development with simultaneous reduction of hazards presented to humans and environment as well as with securing safe high quality agricultural products (it implies strong limitation of toxic pesticides and herbicides). Application of biostimulators seems to be the best means to appease an urgent need for alternative organic methods based on new biologically active, environmentally friendly, and safe substances.

## Biostimulators

Plant biostimulators – phytostimulators – are various non-toxic substances mostly of natural origin that improve and stimulate plant life processes differentially than fertilizers or phytohormones. Their influence on plants is not the consequence of their direct ability to regulate metabolism and their action could be multidirectional. The crucial point is that biostimulators in contrast to bioregulators and hormones improve plant metabolic processes without changing their natural pathway (**Table 1**).

**Table 1:** Bioregulators and biostimulators: general comparison of their properties.

Bioregulators	Biostymulators
Phytohormones	Phytostimulators
Auxins, giberelins, cytokinins, ABA, JA, ethylene, and brassinosteroids	Different substances of natural origin (could be also syntezised), their mixtures, bio-extracts

• Natural (usually secondary metabolites) or synthetic substances (hormone analogs)	• Non-toxic, safe for human and environment substances
• Not nutritional elements	• Supply ready for use beneficial elements or organic compounds that are usually generated via many complicated biochemical processes in plants – time and energy saving
• Transported from the place of their synthesis to the action site in plants	• Active at the site of their absorbtion an/or transpodted all over a plant
• Act at low concentrations	• Act at different concentrations
• Regulate directly plant metabolism at molecular, cytological levels as well as in a whole plant – regulate plant growth and development	• Indirectly regulate life processes influencing metabolism in many ways (**Figure 1**)
• Show pleiotropic effects and often act as signaling molecules responsive to internal and external stimuli	• Some of them are able to influence plant signaling cascades
• Improve plant life processes; however, exogenous application of phytohormons can modify natural pathways of plant development (e.g., induction of fruit parthenogenesis, callus cultures or plant cell culture *in vitro*)	• Improve plant growth and development; rationalize plant life processes not modyfiying their natural program

They benefit plant productivity by interacting with plant-signaling cascades thereby reducing negative plant reactions to stress (Brown and Saa, 2015). Unfortunately, the precise mechanisms activated by biostimulators are difficult to identify because of the complexity of the applied extracts and wide range of molecules contained in the solution (Bulgari et al., 2015). For instance, protein hydrolysates may promote nitrogen (N) assimilation in plants via a coordinated regulation of carbon (C) and N metabolism, as it was found in maize plants supplemented with protein hydrolysate derived from alfalfa plants (Schiavon et al., 2008). There are also some studies concerning the effects of biostimulators on secondary metabolism. Biostimulators derived from agro-industrial by-products improved plant productivity triggering the expression of *ZmPAL* and enhancing L-phenylalanine ammonia lyase (PAL, a key enzyme of phenylpropanoids synthesis) activity in maize leaves (Ertani et al., 2011). Studies of Pardo-García et al. (2014) revealed that oak extract acted as a biostimulator of grape polyphenol synthesis, *i.a*.: gallic acid, hydroxycinnamoyl tartaric acids, acylated anthocyanins, flavanols, and stilbenes. Biostimulators often increase chlorophyll content, which is crucial for proper course of photosynthesis. It was observed in cowpea seeds pre-soaked with a carrot extract (Abbas and Akladious, 2013) and in rocket (*Eruca sativa*) treated with *Moringa oleifera* extract (Abdalla, 2013).

Synthetic pesticides effectively kill the plant pests; however, they are often toxic also for useful insects, may accumulate in the environment and can reduce the quality and safety of agricultural products. This provokes an urgent need to elaborate alternative safe ecological methods. There is a tendency in plant protection toward broader use of biological methods based on non-toxic plant originating substances, having different mode of action. Contrary to pesticides they are usually not directly active against harmful organisms, but they induce in plants certain immunity/resistance to pathogens. The results of Goëmar Labs showed that algae filtrates from *Ascophyllum nodosum* stimulated growth and nutrition of the treated plants and laminarin extracted from *Laminaria digitata* induced natural defense reactions (Joubert and Lefranc, 2008). The studies of their mode of action showed that these products acted as phytoactivators. First, the filtrates stimulated the nitrate reductase and root phosphatases, involved in both N and phosphorus nutrition. Such stimulation resulted in better plant growth and increased chlorophyll content. Moreover, algae homogenate increased free polyamine content in plant tissues, which is important for better fruit harvesting. Second, laminarin is a natural β-1,3-1,6 glucan extracted from brown alga, and it is known that, some of the fungal β-glucans can be involved in the plant defense mechanisms functioning as activators of natural plant resistance. Considering the structural similarity between laminarin and fungal β-glucans, the potential of laminarin to elicit a cascade of natural defense responses causing plant resistance against phytopathogens was studied. Indeed, laminarin is clearly devoid of any direct antimicrobial activity but it induces resistance by plant defense activation (Joubert and Lefranc, 2008). Also betaines act as typical elicitors, factors which induce systemic acquired resistance to pathogens or other stresses in plants. Some biostimulators can also stimulate endophytic and non-endophytic bacteria, yeast and fungi to produce molecules of benefit to plants (Brown and Saa, 2015). Thus, some biostimulators can elicit resistance in plants reducing thereby the need of conventional treatments with synthetic chemicals. They can also possess antimicrobial properties and/or act as insect repellents, e.g., many polyphenols.

Plant supplementation with beneficial elements or organic compounds ready for use, e.g., aminoacids (Cambri, 2008) including proline (Posmyk and Janas, 2007) and the above-mentioned polyphenols (Posmyk et al., 2009b) allow plants to saves energy for other needs such as recovery processes after stress.

Various stresses inhibit plant growth via different mechanisms but all of them cause increase in reactive oxygen species (ROS) content. Over-production of these reactive compounds not followed by their neutralization, disturbs redox homeostasis and induces oxidative stress, which is a well known secondary effect of all biotic and abiotic stresses. Thus, on the one hand antioxidant activity is very desirable in stressed plant cells, on the other hand, it is worth to remembering ROS and free radicals could be also formed naturally as products of biochemical reactions involved in normal metabolic functions (e.g., cellular respiration, photosynthesis, cell-wall biosynthesis, and detoxification processes) and they also play important signaling functions in plants, thus they cannot be eliminated but only restricted.

Phenolic compounds, as effective antioxidants at low concentrations, influence lots of biochemical and physiological processes in plants (Giada, 2013). Components of Asahi SL, a commercially available mixture of sodium *ortho*- and *para*-nitrophenolates with sodium 5-nitroguaiacolate, are easily metabolized by plants into other phenolics which are involved in mitochondrial energy generating processes. Their action results also in low cytoplasm viscosity, which fosters translocation of all biosynthesis products. Moreover, diphenols are specific inhibitors of auxin oxidases so indirectly they have positive influence on this important phytohormone activity.

Being antioxidants, e.g., anthocyanins (ATH; Posmyk et al., 2009b) or osmoprotectants, e.g., proline (Posmyk and Janas, 2007) biostimulators can provoke plant tolerance of unfavorable environmental conditions such as chilling, drought, salinity, chemical contamination, and heavy metal stresses. The results of Posmyk et al. (2009b) revealed the role of ATH-rich cabbage extract in the protection of *Vicia faba* root meristematic cells against copper stress (Cu^2+^). The surprisingly good effect of ATH influence was observed even after Cu^2+^ treatment. This may suggest that ATH not only significantly limits, but heals cytological disturbances caused by Cu^2+^ as well. Generally, ATH applied before and during Cu^2+^ stress protected root meristems against toxic heavy metal activity preventing genetic damage. The mechanisms of their action still need elucidation. Evidence suggests that polyphenols including flavonoids (e.g., ATH) can act in one or more ways: (i) modulating activation of metabolic mutagens, (ii) by stimulating antioxidant and/or detoxification enzymes, (iii) directly scavenging free radicals [(ii) and (iii) limit secondary oxidative stress therefore inhibit lipoprotein and lipid peroxidation], (iv) forming strong ligand complexes with ions of transition metals, such as Fe, Cu, and Mo (their chelating properties lead to metal isolation in epidermis, cell wall or vacuoles). The phenomenon of DNA structure stabilization by ATH is also widely discussed in literature (Lazze et al., 2003).

It was also shown (Posmyk and Janas, 2007) that pre-sowing proline application into seeds increased germination rate at low temperature and protected susceptible *Vigna radiata* seedlings against chilling injury. There is a lot of information that proline acting as an osmolyte plays an important role in membrane stabilization and it seems to be involved in biosynthesis of phenolics which scavenge free radicals (Shetty, 2004). It also facilitates quicker repair of injuries caused by stress probably being an alternative source of N and C, improving seedling growth and regeneration (Posmyk and Janas, 2007).

Our research group was the first that supplemented seed priming with melatonin as an exogenous biostimulator. Our experiments with *Brassica oleracea*, *Cucumis sativus*, *V. Radiata*, and *Zea mays* (Posmyk et al., 2008, 2009a; Szafrańska et al., 2013, 2014; Kołodziejczyk et al., 2015) proved that exogenous melatonin applied into seeds by pre-sowing treatment improved their vigor and germination as well as seedling growth even under adverse environmental conditions.

The latest results (Kołodziejczyk et al., 2016). indicated that melatonin seed treatment expediently modified proteome of maize (*Z. mays* L.) embryo during seed germination. The majority of additional proteins were (i) energy metabolism enzymes, (ii) proteins involved in proteome plasticity via improving protein synthesis, folding, destination and storage, and – most importantly – (iii) defense, anti-stresses, and detoxifying proteins. This explains why the seeds hydroprimed with melatonin and the seedlings grown from them were stronger in comparison to the non-treated ones, and so quickly and efficiently adapted to changing environmental conditions. They were *a priori* prepared to cope with potential harmful conditions. These results partially explain how melatonin acts in plant stress defense and why various plant species rich in melatonin have shown higher capacity for stress tolerance (Zhang et al., 2015). Further research in this area could provide valuable information to improve organic farming and environmental phytoremediation; however, already the presented knowledge indicate that melatonin could be also used as an effective biostimulator (Janas and Posmyk, 2013; Kołodziejczyk and Posmyk, 2016).

## Application Time and Methods

Phytostimulators are usually used as supplementation of irrigation or *in substantia* with fertilizers into roots and also with foliar fertilizers or protection spraying. They could be also added to the medium in hydroponical cultivation (Glińska et al., 2007). The moment of biostimulator application is very important, they should be used at plant development stages crucial for prospective yield quality and quantity, e.g., during young seedlings sprouting, flowering, and fruit setting (preventive method). Biostimulators are also recommended as an intervention method to be used in case of stress conditions, e.g., black frost, drought, hail, strong wind, and chemical contamination with herbicides or pesticides. They can be applied before expected stress, during unfavorable condition and also after stress for better plant recovery (Glińska et al., 2007).

The quality of seed material is a primary and basic criterion determining good yields. Thus finding effective methods to improve sowing material by applying biostimulators into seeds is a crucial problem. For that reason the known techniques of seed priming: hydro- and osmo-conditioning were tested by the group of Posmyk et al. (Posmyk and Janas, 2007; Posmyk et al., 2008, 2009a) Generally, pre-sowing seed treatments effectively counteract diseases and pests as well as improve seed viability and seedling vigor *per se* (Jisha et al., 2013). All mentioned techniques are based on controlled seed hydration and subsequent re-drying. They can be combined with other supporting factors such as aeration, light-irradiation, and temperature-stratification. Seed priming can also be combined with application of growth regulators and other bioactive substances. Generally, priming is accompanied by an increase in the activities of numerous enzymes, e.g., phosphatases, synthases, peroxidases, and other antioxidant enzymes (Jisha et al., 2013). Intensification of protein and nucleic acid metabolism is also observed (Kołodziejczyk et al., 2016). Moreover, priming facilitates cell membrane structure reorganization from hexagonal (in dry seeds) to lamellar (in imbibed seeds). This reorganization is necessary for biophysical and biochemical processes during germination and accelerates seedling emergence (Jisha et al., 2013). Priming allows plants to survive environmental stresses since it improves their recovery potential and its positive effects could be intensified by additional application of hydrophilic biostimulators as it was mentioned above.

## Conclusion

**FIGURE 1:**
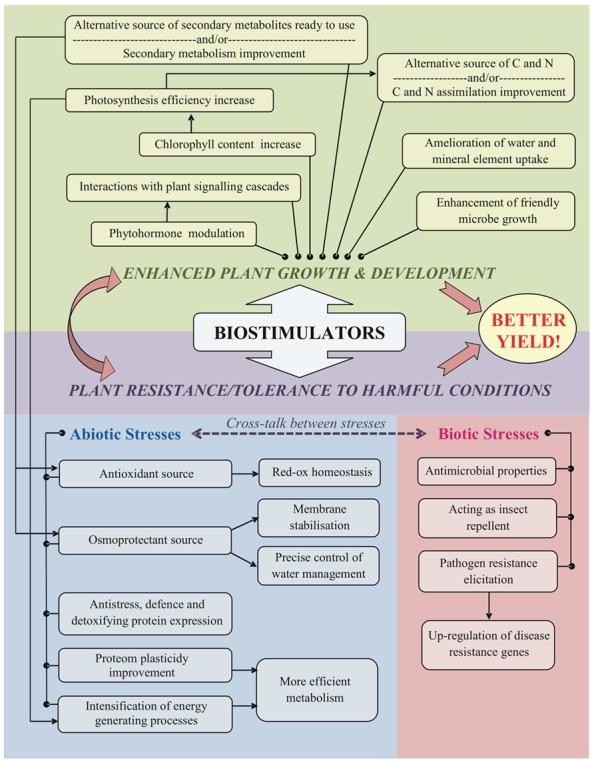
**Biostimulator modes of action and their correlations**.

Nowadays, biostimulators are used in agriculture, floriculture and horticulture, on vegetable plantations and in orchards. There are commercially available synthetic mixtures of phenolic compounds, e.g., Asahi SL or Atonik SL, but also natural ecologically preferable substances can be found such as plant extracts, e.g., Adbios 850 SL – the mixture of oxyethyleneamines from coconut oil and methylesters from canola oil, alga extracts, e.g., AlgaminoPlant – an extract from brown alga supplemented with aminoacid composition; or complexes of humic and fulvic acids extracted from laminarite – HumiPlant. Precise mechanisms of biostimulator actions are difficult to define due to their diversity and/or complexity, but some important pathways have been indicated on **Figure 1**.

To sum up, an ideal biostimulator should have the following characteristics it has to be (i) non-toxic, safe for animals and environment; (ii) easily and actively taken up by plants from environment; (iii) of natural origin or easily synthesized in laboratories; (iv) not expensive; (v) dissolved in different solvents: water, alcohols but also lipids – that facilitates the use of various application methods; (vi) easily penetrating cell compartments, (vii) it must improve plant resistance to adverse conditions and help generate tolerance to stresses.

Many scientists as well as breeders consider new biostimulator searching and application as the most prospective and promising method to produce ecological crops, to protect the environment, and to support safety-food production.

## Author Contributions

MP: work concept, the manuscript revising, and final approval of the version to be published. KS: drafting of the manuscript.

## Conflict of Interest Statement

The authors declare that the research was conducted in the absence of any commercial or financial relationships that could be construed as a potential conflict of interest.
